# Impact of Chronic Infection on Resistance and Tolerance to Secondary Infection in Drosophila melanogaster

**DOI:** 10.1128/iai.00360-22

**Published:** 2023-02-16

**Authors:** Abigail M. Wukitch, Madyline M. Lawrence, Francesco P. Satriale, Alexa Patel, Grace M. Ginder, Emily J. Van Beek, Owais Gilani, Moria C. Chambers

**Affiliations:** a Department of Biology, Bucknell University, Lewisburg, Pennsylvania, USA; b Department of Mathematics, Bucknell University, Lewisburg, Pennsylvania, USA; Universite de Geneve

**Keywords:** chronic infection, Drosophila, Providencia, Serratia, bacterial pathogenesis, innate immunity, superinfection, tolerance

## Abstract

Prior exposure to a pathogen can greatly influence the outcome of a secondary infection, and although invertebrates lack classically defined adaptive immunity, their immune response is still influenced by prior immune challenges. While the strength and specificity of such immune priming depends highly on the host organism and infecting microbe, chronic bacterial infection of the fruit fly Drosophila melanogaster with species isolated from wild-caught fruit flies provides broad nonspecific protection against a later secondary bacterial infection. To determine how chronic infection influences progression of secondary infection, we specifically tested how chronic infection with Serratia marcescens and Enterococcus faecalis impacted both resistance and tolerance to a secondary infection with an unrelated bacterium, Providencia rettgeri, by simultaneously tracking survival and bacterial load postinfection across a range of infectious doses. We found that these chronic infections increased both tolerance and resistance to *P. rettgeri*. Further investigation of S. marcescens chronic infection also revealed robust protection against the highly virulent Providencia sneebia, and that protection was dependent on the initial infectious dose for S. marcescens with protective doses corresponding with significantly increased diptericin expression. While the increased expression of this antimicrobial peptide gene likely explains the increased resistance, increased tolerance is likely due to other alterations in organismal physiology, such as increased negative regulation of immunity or tolerance of ER stress. These findings provide a foundation for future studies on how chronic infection influences tolerance to secondary infection.

## INTRODUCTION

While many infections are studied in isolation in a laboratory setting, infections often occur in a much messier landscape where organisms have previous exposure to both the same and other pathogens. The modulation of immune responses by prior exposure is likely controlled by both adaptive and innate immune mechanisms. Although invertebrates lack the B and T cells associated with adaptive immunity in vertebrates, invertebrates can also respond differently to an infection based on prior exposure and the mechanisms behind this innate immune priming are less understood. Immune priming is seen in a wide range of insects from beetles to fruit flies (1) suggesting that there are potentially some highly conserved mechanisms that invertebrates have evolved to help them combat exposures to multiple pathogens. Specificity and strength of priming, however, vary depending on the experimental system (1, 2).

The fruit fly, Drosophila melanogaster, has been used to study the innate immune system using a wide range of bacterial pathogens, including those that commonly infect humans (e.g., Listeria monocytogenes and Salmonella Typhimurium) and bacterial species isolated from wild-caught flies (e.g., *Providencia* and *Serratia* species) (3, 4). There are three potential outcomes of bacterial infections within the host; the bacteria kill their host, are cleared below the limit of detection, or persist and generate long term chronic infections with minimal impact on host life span (5–7). For three pathogens, Serratia marcescens, Providencia rettgeri, and Enterococcus faecalis, a subset of flies succumbs to infection within a few days depending on the infectious dose, but any fly that survive this initial phase of infection sustain a chronic bacterial infection for the remainder of their life, never clearing the pathogen (5–7). The number of bacteria that persist depends on the infectious dose used to initiate the infection, which provides an opportunity to study how pathogen load during chronic infection affects the host (5, 7).

Flies chronically carrying a moderate to high pathogen load of S. marcescens, *P. rettgeri*, and E. faecalis have improved survival to secondary infections with both the same and unrelated species (7). Similarly, flies chronically infected with a low virulence strain of Pseudomonas aeruginosa are protected against a more virulent P. aeruginosa infection, although it is unknown whether this chronic infection would protect against other species as well (8). However, it remains unclear why these chronically infected fruit flies have such broad protection against secondary infection. As E. faecalis is Gram-positive, while the S. marcescens and *P. rettgeri* are Gram-negative, the protective response is not dependent on Gram-type and likely due to changes elicited during all three infections (4). Flies chronically infected with S. marcescens, *P. rettgeri*, or E. faecalis continue to have increased expression of their antimicrobial peptide (AMP) genes as the chronic infection persists, although the strength of upregulation of specific AMPs depends on the pathogen (4, 7, 9). It is uncertain, however, whether this increased expression is responsible for the improved survival or if other factors like interspecies competition or alterations to host tolerance may be responsible for the protective effects.

Some previous mechanistic studies on priming have used heat-killed or formaldehyde-inactivated bacteria as the initial exposure, but none of these exposures to dead bacteria have provided the broad protective effects seen during chronic infection. Exposure to heat-killed Streptococcus pneumoniae confers highly specific protection and is dependent on phagocytosis (10). Exposure to heat-killed P. aeruginosa results in protection against later infection with P. aeruginosa and depends on the presence of the *imd* pathway (8). Injection of heat-killed Pseudomonas entomophila did not impact survival or fecundity after later infection with *P. entomophila*, but did improve the ability to resist the infection as measured through bacterial load postinfection (11). Additionally, injection of heat-killed bacteria does not always induce priming. Injection of heat-killed Lactococcus lactis, Providencia burhodogranariea, Mycobacterium *marium*, Listeria monocytogenes, and Salmonella Typhimurium all failed to produce protection against later infection (12). This suggests that either the type or the strength of the changes induced by chronic infection (or both) are different from those induced through injection of dead bacteria. For this reason, it is necessary to investigate responses to a pathogen while a previous infection is still ongoing.

Determining protection mechanisms during two ongoing infections is complicated. Selectively inhibiting immune responses impacts both infections and may prevent the organism from surviving and sustaining chronic infection. One way to distinguish between the plethora of possible mechanisms responsible for this protection is to first learn whether chronic infection improves resistance, tolerance, or both. Resistance is the ability of the host to kill the pathogen or constrain the pathogen's ability to grow within the host resulting in the limitation of overall pathogen number. Tolerance is the ability of the host to minimize the negative effects of the pathogen’s presence through things like regulation of metabolism, wound healing and negative regulation of the host’s own immune response (13–16). The relative importance of these two factors can be determined by simultaneously monitoring changes in health and changes in pathogen number during the infection and then determining how this relationship is altered under various conditions (13, 17–20). If conditions largely change pathogen number, but the relationship between health and pathogen load remains consistent, then resistance is likely the dominant change. If the relationship between pathogen number and health is altered, then it suggests that tolerance is important. There is growing recognition that the relationship between pathogen load and health is best modeled using nonlinear regression, which allows researchers to distinguish between multiple types of shifts in tolerance, which may suggest different underlying mechanisms (17, 20). By disentangling these mechanisms, we may better understand how the innate immune system, in the absence of an adaptive response, can modulate responses to future infections.

In this paper, we further characterized the protective effect of chronic infection during secondary infection in D. melanogaster, finding that chronic infection with S. marcescens or E. faecalis improved both tolerance and resistance to the unrelated pathogen *P. rettgeri* suggesting that protection is due to multiple mechanisms. We found that the protective effect of S. marcescens is particularly robust and persists at a wide range of infectious doses and even protects against the highly virulent *Drosophila* pathogen Providencia sneebia.

## RESULTS

### Chronic infection with S. marcescens and E. faecalis protects against secondary infection with *P. rettgeri* across a range of doses.

One mechanism for assessing the impact of tolerance is to assess both bacterial load and survival postinfection at a range of infectious doses across conditions of interest (17, 20). To determine whether chronic infection reduced mortality following secondary infection at a range of infectious doses, flies were all given the same dose of bacteria via abdomen injection to establish chronic infection, and then, one week later, secondary infections with a different bacterial species through thorax injection. Statistical significance was determined by first performing a log-rank test on flies from a single experiment comparing flies carrying both chronic infection and secondary infection with flies carrying only a secondary infection at the same dose. All doses were assessed through independent replicates on at least three different dates, and the *P* values from all the dates were combined using Fisher’s method as long as effects were in the same direction from data collected on every date (see Table S1 and S2 in the supplemental material).

**FIG 1:**
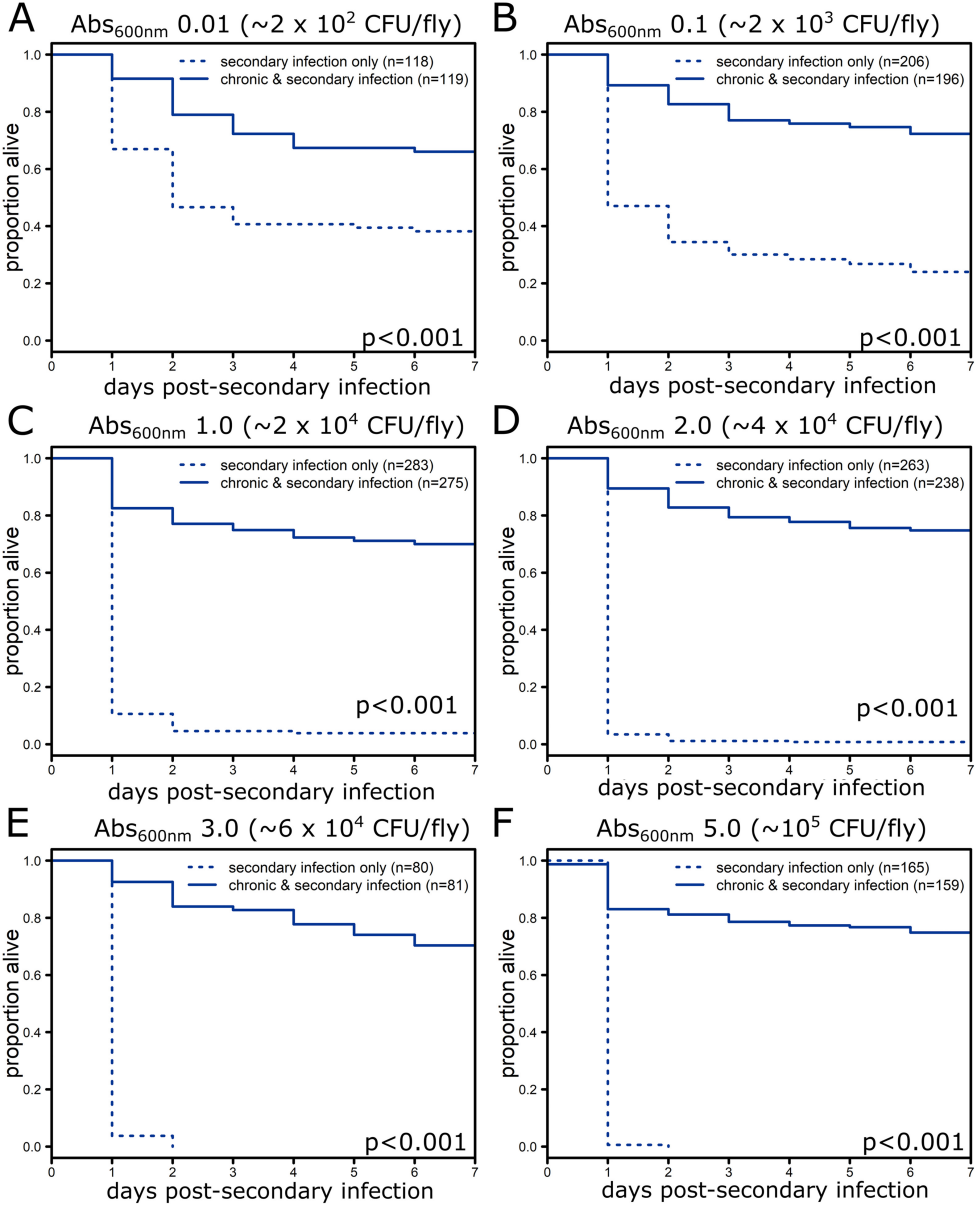
Chronic infection with S. marcescens protects against *P. rettgeri* secondary infection at a range of doses. Five to seven day old flies were injected in the abdomen with either sterile phosphate-buffered saline or a bacterial suspension of S. marcescens (Abs_600nm_0.1, approximately 3 × 10^3^ CFU/fly) to initiate chronic infection and then, one week later, injected with a bacterial suspension of *P. rettgeri* to initiate secondary infection at one of six concentrations ranging from 2 × 10^2^ to 10^5^ CFU/fly: Abs_600nm_ 0.01 (A), 0.1 (B), 1.0 (C), 2.0 (D), 3.0 (E), or 5.0 (F). Significance was determined by performing log-rank tests of flies injected on the same day comparing flies carrying both chronic infection and secondary infection with those carrying only secondary infection at the same dose. Since all individual experiments showed improved survival with chronic infection, the *P* values were combined using Fisher’s method (Table S1). Graphs show mortality data (combined across all days) from experiments at each dose; total number of flies per condition is indicated in the legends, while the combined *P* value from Fisher’s method is reflected on each panel.

Chronic infection with S. marcescens provided protection against mortality following secondary infection with *P. rettgeri* at all infectious doses tested relative to flies that only received a secondary infection (Fig. 1, Table S1; *P* < 0.001). Even with infectious doses that caused complete mortality in control flies within 7 days postinjection, flies chronically infected with S. marcescens have less than 30% mortality over the same time frame (Fig. 1E and F). Flies chronically infected with S. marcescens and subsequently injected with sterile saline had minimal mortality (Fig. S1).

**FIG 2:**
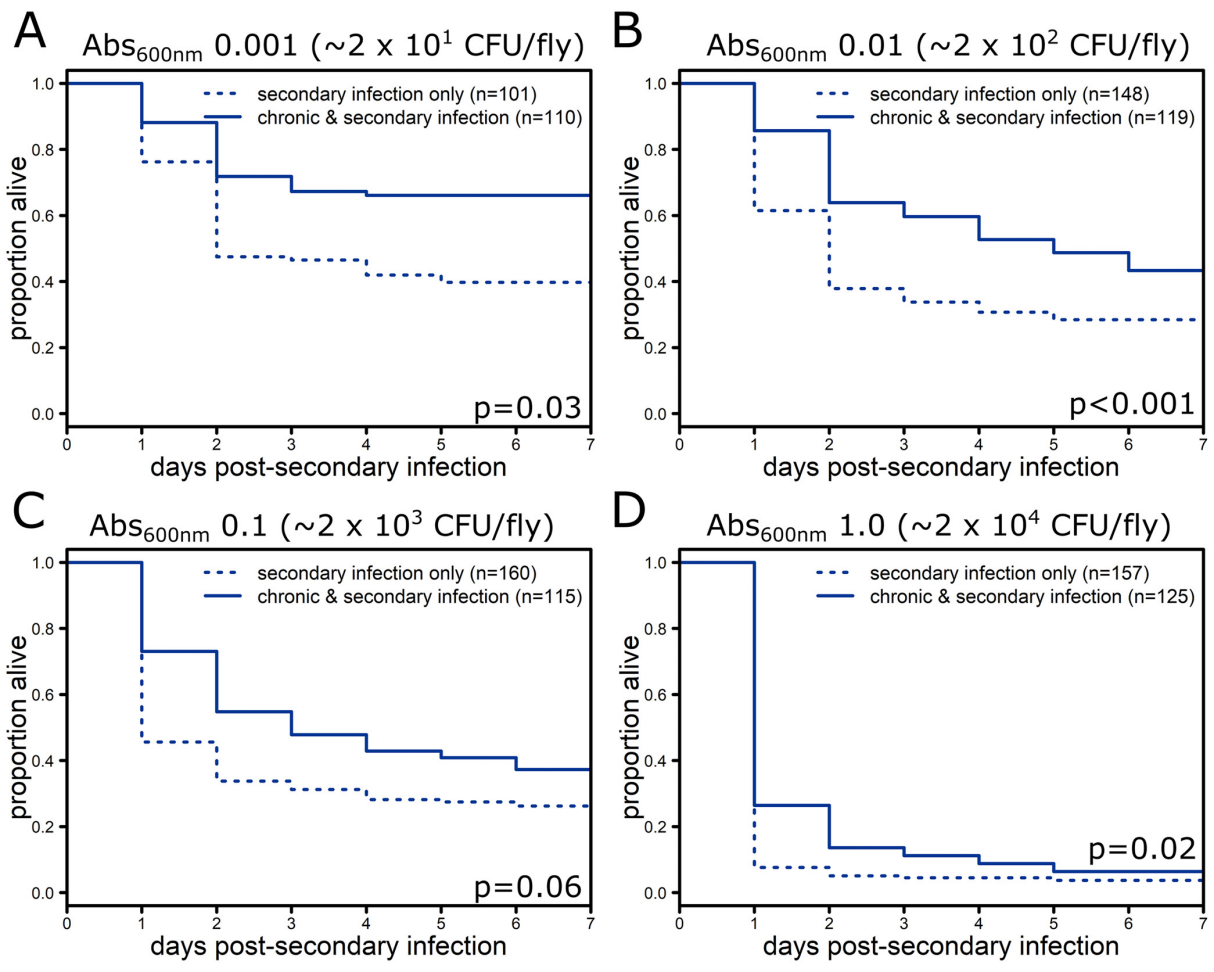
Chronic infection with E. faecalis protects against *P. rettgeri* secondary infection at a subset of doses. Five to seven day old flies were injected in the abdomen with either sterile phosphate-buffered saline or a bacterial suspension of E. faecalis (Abs_600nm_ 0.1, approximately 2 × 10^3^ CFU/fly) to initiate chronic infection and then, one week later, injected with a bacterial suspension of *P. rettgeri* to initiate secondary infection at one of four concentrations ranging from 20 to 2 × 10^4^ CFU/fly: Abs_600nm_ 0.001 (A), 0.01 (B), 0.1 (C), or 1.0 (D). Significance was determined by performing log-rank tests of flies injected on the same day comparing flies carrying both chronic infection and secondary infection with those carrying only secondary infection at the same dose. Since all individual experiments showed improved survival with chronic infection, the *P* values were combined using Fisher’s method (Table S2). Graphs show mortality data (combined across all days) from experiments at each dose; total number of flies per condition is indicated in the legends, while the combined *P* value from Fisher’s method is reflected on each panel.

Chronic infection with E. faecalis provided protection against secondary infection with *P. rettgeri* at three out of the four doses tested (Fig. 2, Table S2; *P* < 0.05). The one dose that did not yield significant protection (Abs_600nm_ = 0.1) had a borderline *P* value (Fig. 2C; *P* = 0.06). The magnitude of this protective effect was also smaller than seen with S. marcescens with only 5 to 20% more chronically infected flies surviving relative to control flies that received only the secondary infection (Fig. 2).

### Chronic infection with S. marcescens and E. faecalis alters resistance and tolerance to secondary infection with *P. rettgeri*.

To assess whether this protection was due to changes in resistance and/or tolerance, the *P. rettgeri* bacterial load was measured 10 h post-secondary infection in a subset of flies and survival 3 days postinfection was monitored for remaining flies injected on the same day as this is the time frame in which most mortality from *P. rettgeri* infection occurs (4, 20–22).

**FIG 3:**
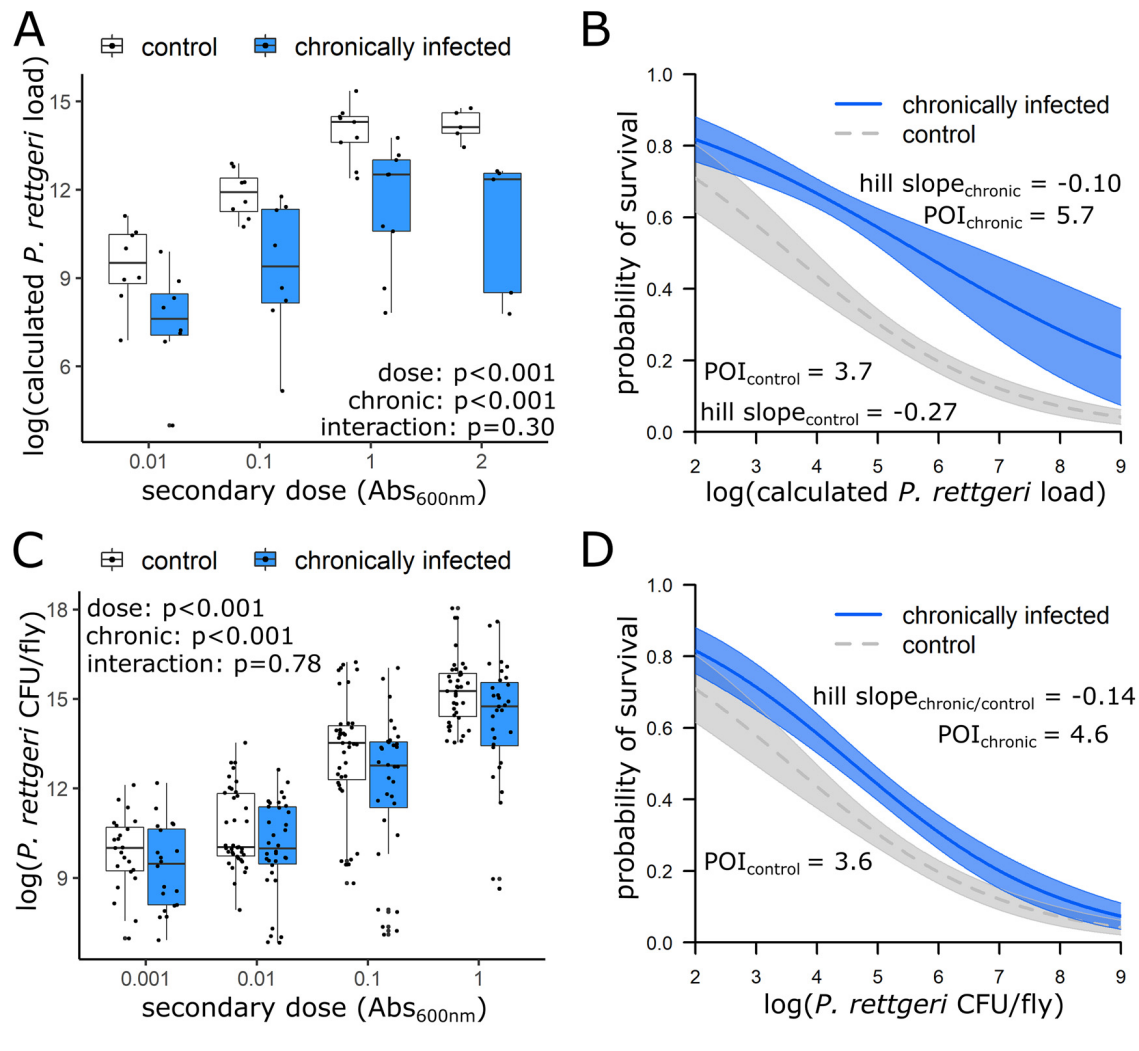
Chronic infection with S. marcescens and E. faecalis impact resistance and tolerance to *P. rettgeri* secondary infection. Five to seven day old flies were injected with either sterile phosphate-buffered saline or a bacterial suspension of S. marcescens (A and B) or E. faecalis (C and D) (Abs_600nm_0.1, approximately 2 × 10^3^ CFU/fly) in the abdomen to initiate chronic infection and then, 1 week later, injected with a bacterial suspension of *P. rettgeri* at one of five concentrations ranging from approximately 20 to 4 × 10^4^ CFU/fly: Abs_600nm_ 0.001, 0.01, 0.1, 1.0, or 2.0. Bacterial load of *P. rettgeri* was determined at 10 h post-secondary infection using either (A) qPCR or (C) a CFU assay. Survival of remaining flies at three days postinfection was used to build logistic regression models. Output of logistic regression models are depicted for (B) S. marcescens chronic infection and (D) E. faecalis chronic infection. POI, point of inflection.

Changes in resistance were determined by comparing the *P. rettgeri* bacterial loads between chronically infected and control flies 10 h postinfection. Bacterial loads were assessed at 10 h postinfection, as this time point allowed sufficient bacterial replication to detect differences in resistance but is before any flies begin to die from infection (5, 7, 21). Flies chronically infected with S. marcescens or E. faecalis had significantly lower *P. rettgeri* loads 10 h postinfection, indicating improved resistance (*P* < 0.001; Fig. 3A and C). For both S. marcescens and E. faecalis infection, there was insufficient evidence that the impact of chronic infection on bacterial load differed across the doses of the secondary infection (*P* = 0.30 and *P* = 0.78, respectively).

Changes in tolerance were determined through logistic regression models with probability of survival at 3 days postinfection as the response variable and log_10_ (bacterial load) at 10 h postinfection, chronic infection status, and the interaction between the two, as the explanatory variables (Table 1). By examining the main effect of chronic infection, we determined whether chronic infection improved tolerance by shifting the whole curve to the right, or reduced tolerance by shifting the curve to the left. Through examination of the interaction between chronic infection and log_10_ (bacterial load) we determined if chronic infection altered the slope of the relationship between log_10_(bacterial load) and probability of survival. For chronic infections with S. marcescens, there was both a significant shift of the curve to the right (*P* = 0.02) and change in the slope (Fig. 3B; *P* < 0.001). The point of inflection, where the probability of survival is 50% at 3 days post-secondary infection, shifted 2 log_10_(CFU equivalents/fly) to the right: moving from 5 × 10^3^ CFU equivalents/fly for control flies with only a secondary infection to 5 × 10^5^ CFU equivalents/fly for flies carrying both chronic infection with S. marcescens and secondary infection with *P. rettgeri* (Fig. 3B). The Hill slope, which is the slope at the point of inflection, was significantly steeper for control flies (−0.27) relative to the slope for flies chronically infected with S. marcescens (−0.10), suggesting that the cost of higher bacterial loads is less for chronically infected flies relative to controls (Fig. 3B).

**TABLE 1:** Output of logistic regression for impact of bacterial load and chronic infection on survival 3 days post-secondary infection

	Chronic infection					
	S. marcescens			E. faecalis		
Factor	Estimate	Z value	*P* value	Estimate	Z value	*P* value
(Intercept)	3.93	0.59	<0.001	2.04	0.36	<0.001
Log_10_ (bacterial load)	−1.06	−8.66	<0.001	−0.57	−8.87	<0.001
Chronic infection	−1.62	−2.32	0.02	0.60	4.47	<0.001
Log_10_ (bacterial load):chronic infection	0.66	4.40	<0.001			

For E. faecalis chronic infection, the full model showed no significant impact of chronic infection on probability of survival (Table S3). The interaction term was also not statistically significant (*P* > 0.05), suggesting that the slope was not different across the conditions. We therefore removed the interaction term from the model. The simpler model revealed a significant effect of chronic infection (*P* < 0.001), shifting the survival curve to the right (Fig. 3D). The point of inflection shifted 1 log_10_ (CFU equivalents/fly) to the right from 4 × 10^3^ CFU per fly for control flies with only a secondary infection to 4 × 10^4^ CFU equivalents per fly for flies carrying both chronic infection with E. faecalis and secondary infection with *P. rettgeri*. The Hill slope was −0.14 for both control flies and chronically infected flies.

### Chronic infection with S. marcescens improves survival during secondary infection with *P. sneebia*.

**FIG 4:**
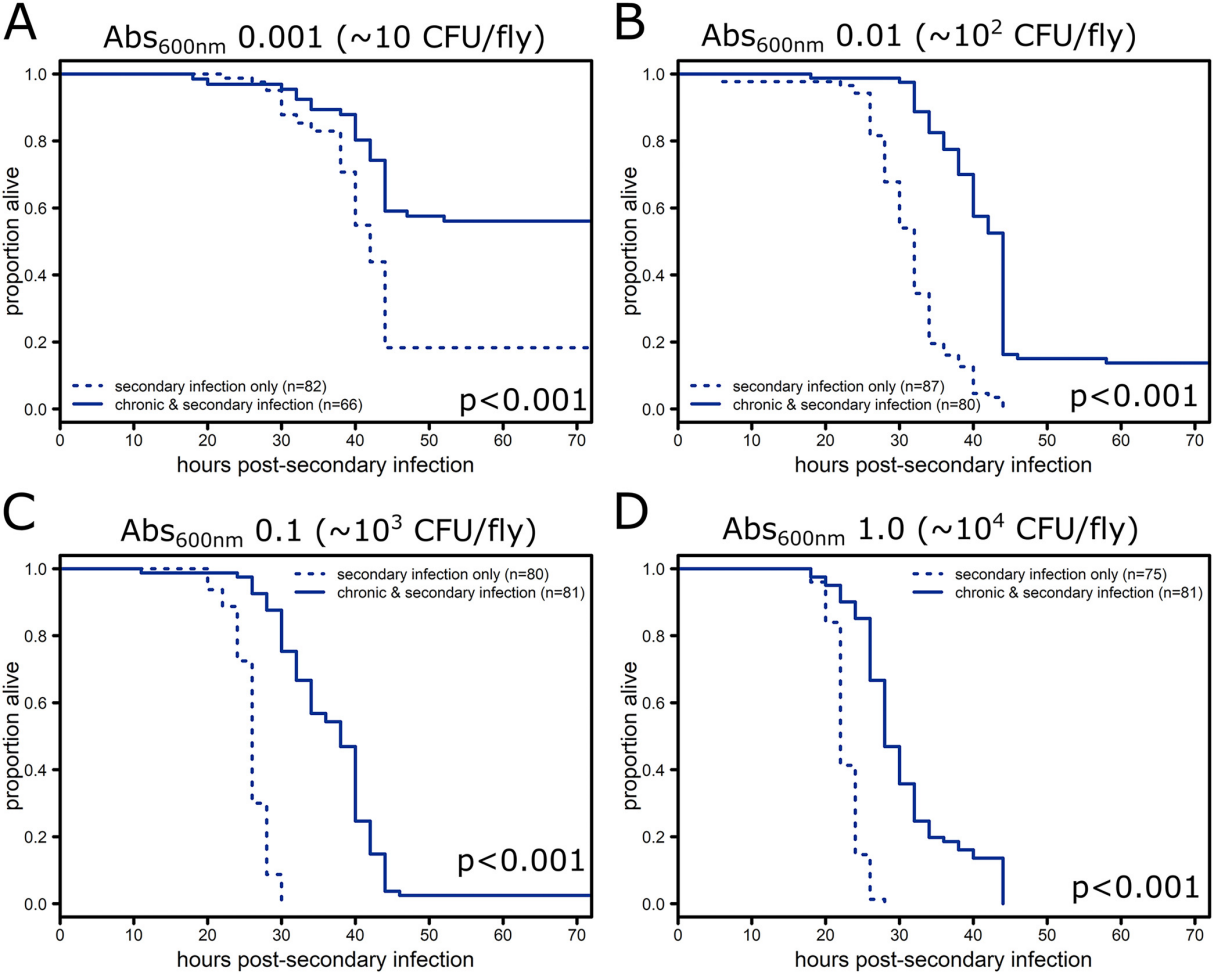
Chronic infection with S. marcescens protects against *P. sneebia* secondary infection. Five to seven day old flies were injected in the abdomen with either sterile phosphate-buffered saline or a bacterial suspension of S. marcescens (Abs_600nm_ 0.1, approximately 2 × 10^3^ CFU/fly) to initiate chronic infection and then, one week later, injected with a bacterial suspension of *P. sneebia* to initiate secondary infection at one of four concentrations ranging from approximately 10 to 10^4^ CFU/fly: Abs_600nm_ 0.001 (A), 0.01 (B), 0.1 (C), or 1.0 (D). Significance was determined by performing log-rank tests of flies injected on the same day comparing flies carrying both chronic infection and secondary infection with those carrying only secondary infection at the same dose. Since all individual experiments showed improved survival with chronic infection, the *P* values were combined using Fisher’s method and the final combined *P* value is reflected on each panel (Table S3). Graphs show combined mortality data from experiments at that dose and total number of flies per condition is indicated in the legends.

To further distinguish potential pathways responsible for protection, we focused our efforts on chronic infection with S. marcescens, because this chronic infection had more robust protection against secondary infection. To determine whether chronic infection with S. marcescens would provide protection against a more virulent pathogen, flies chronically infected with S. marcescens were given a secondary infection with *P. sneebia*, a pathogen that does not elicit a significant increase in antimicrobial peptide (AMP) gene expression and typically results in complete mortality even at doses as low as 30 to 50 CFU/fly (20, 22). Chronically infected flies and control flies, which were initially injected with sterile phosphate-buffered saline, were secondarily injected with *P. sneebia* at four doses ranging from (Abs_600nm_ 0.001 to 1.0, approximately 10 to 10^4^ CFU/fly) resulting in complete mortality in control flies for the three highest doses and greater than 80% mortality for the lowest dose 3 days postinfection (Fig. 4). Chronic infections with S. marcescens provided significant protection from mortality following highly lethal secondary infections at all infectious doses tested (Fig. 4, Table S4; *P* < 0.0001). Flies chronically infected with S. marcescens had less than 20% survival 3 days postinfection with the three highest doses of *P. sneebia* (Fig. 4B–D), but at the lowest infectious dose there was more than 50% survival 3 days postinfection (Fig. 4A).

### Moderate infectious doses of S. marcescens provide consistent protection against secondary infection with *P. rettgeri* and *P. sneebia*.

**FIG 5:**
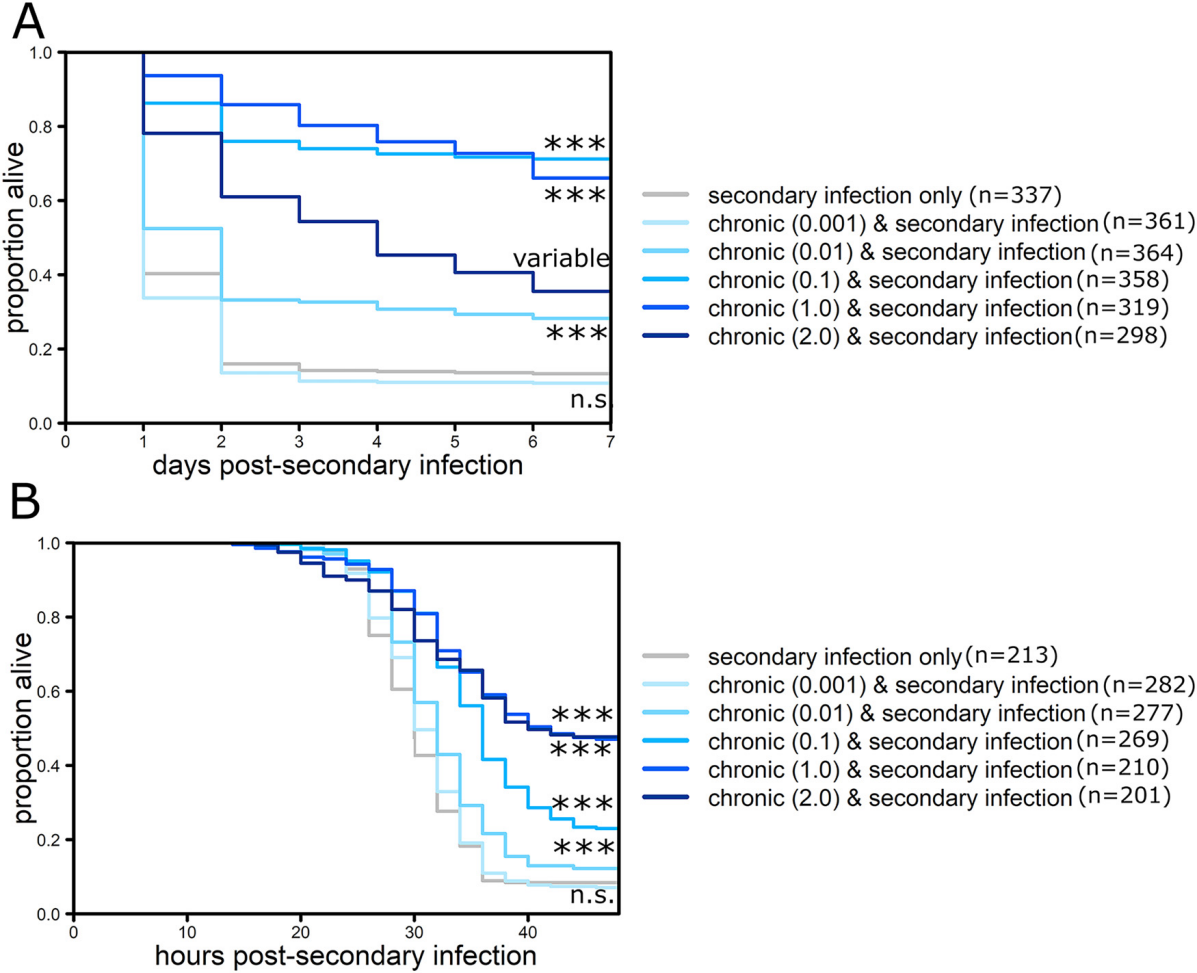
Infectious dose of chronic infection influences protection during secondary infection. Five to seven day old flies were injected with a bacterial suspension of S. marcescens at one of five different doses ranging from 20 to 40 × 10^4^ CFU/fly (Abs_600nm_ 0.001, 0.01, 0.1, 1.0, or 2.0) to initiate chronic infection or sterile phosphate-buffered saline solution in the abdomen and then, one week later, injected with (A) *P. rettgeri* (Abs_600nm_ 2.0, approximately 6 × 10^4^ CFU/fly) or (B) *P. sneebia* (Abs_600nm_ 0.01, approximately 10^2^ CFU/fly). Statistical significance was determined by performing log-rank tests of flies injected on the same day comparing each chronic infection and secondary infection condition with those carrying only secondary infection and combining those *P* values using Fisher’s method, if applicable (Table S4 and S5). Statistical significance is indicated on the graph: ***, *P* < 0.001; n.s., not significant; variable, could not combine *P*-values from individual experiments using Fisher’s method. Graphs show combined mortality data for all experimental dates and total number of flies per condition is indicated in the legends.

The number of bacteria present during chronic infection could both influence interspecies competition during the secondary infection and influence the level of AMP gene expression, which correlates with bacterial number during other infections (8, 16). To determine whether the number of bacteria in the initial infection influenced protection, chronic infections were established with various doses of S. marcescens which causes a corresponding range of chronic infection bacterial loads (5, 7). Moderate infectious doses (2 × 10^2^ to 2 × 10^4^ CFU/fly) provided significant protection against secondary infection with both *P. rettgeri* and *P. sneebia* (*P* < 0.001; Fig. 5, Table S5 and 6). The lowest infectious dose of S. marcescens (Abs_600nm_ 0.001) did not provide significant protection for either *P. rettgeri* or *P. sneebia* secondary infection (*P* = 0.76 and 0.26, respectively; Fig. 5, Table S5 and S6). The highest dose (Abs_600nm_ 2.0) provided significant protection during secondary infection with *P. sneebia* (*P* < 0.001; Fig. 5B, Table S6). During secondary infection with *P. rettgeri*, however, the highest dose of S. marcescens provided variable protection across experiments completed on eight different dates: two dates demonstrated significant protection (*P* < 0.001), while five dates had impacts on survival consistent with protection but not significant in isolation (*P* > 0.05), and on one date the survival was flipped with chronically infected flies doing worse than controls. Therefore, we were unable to combine tests from individual dates to make a more generalized conclusion about the protection offered by the highest dose of S. marcescens against secondary infection with *P. rettgeri*.

### Doses of S. marcescens that confer protection have elevated *diptericin* expression.

**FIG 6:**
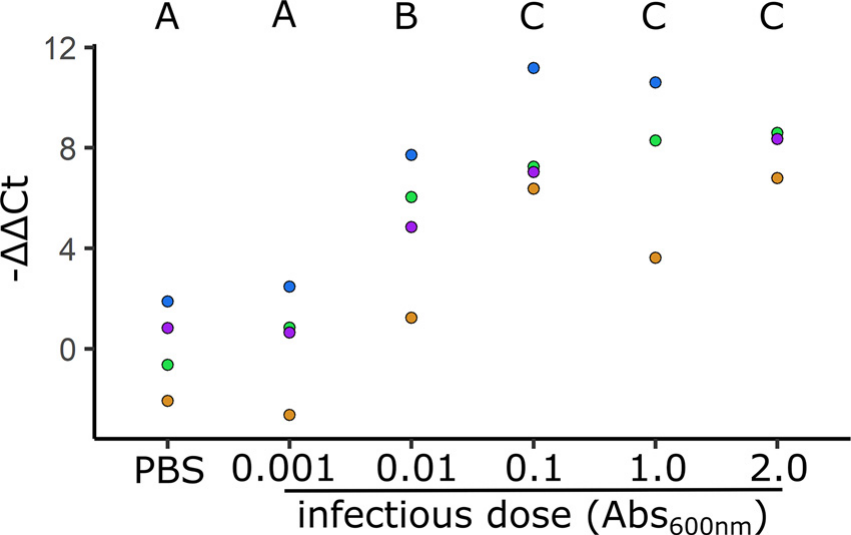
Impact of infectious dose on *diptericin* expression during chronic infection. Five to seven-day-old male flies were injected in the abdomen with sterile saline or a bacterial suspension of S. marcescens at one of five different doses ranging from 20 to 40 × 10^4^ CFU/fly (Abs_600nm_ 0.001, 0.01, 0.1, 1.0, or 2.0) to initiate chronic infection and then RNA was isolated 1 week later. Relative *diptericin* expression was determined using qRT-PCR and analyzed using the ΔΔCt method using Rp49 as a housekeeping gene and the flies injected with sterile saline solution as a control condition. Impact of dose on *diptericin* expression was determined using an ANOVA followed by a Tukey test. Treatments that are not statistically different (*P* > 0.05) share a capital letter above, doses that do not share the same letter had statistically significant differences (*P* < 0.05). Samples with the same fill color were generated on the same day.

Since flies initially infected with the lowest dose of S. marcescens did not exhibit any protection during secondary infection, we tested whether flies infected with this dose had correspondingly lower expression of *diptericin*, an antimicrobial peptide that has a large impact on survival during *P. rettgeri* infection (23). Flies were injected with S. marcescens ranging from 0.001 to 2.0 Abs_600nm_ or approximately 30 to 6,000 CFU per fly, and expression 1 week postinfection determined using qRT-PCR. Flies injected with the lowest dose (Abs_600nm_ 0.001) had significantly lower expression than flies infected with the higher doses (*P* < 0.05; Fig. 6). Samples from flies infected with higher doses (Abs_600nm_ 0.01 to 2.0, approximately 300 to 6,000 CFU/fly) expressed *diptericin* 5 to 500-fold more than sterile saline injected control but had no significant differences between the individual doses.

## DISCUSSION

In this paper, we further characterized the protective effect of chronic infection during secondary infection in D. melanogaster, finding that chronic infection with S. marcescens or E. faecalis improved both tolerance and resistance to the unrelated pathogen *P. rettgeri*. While one of these two strategies may dominate in a given scenario, it is also possible for them to change simultaneously. Theory suggests, and some host-pathogen studies find, that there are trade-offs between resistance and tolerance as resistance responses can be costly and cause damage to the host (16, 24, 25). However, there does not appear to be inherent evolutionary constraints requiring this trade-off, as resistance and tolerance sometimes show no relationship or exhibit a positive correlation (19, 22, 26, 27). In the case of chronic infection, it makes sense that the host may have altered responses to make it better at both resisting and tolerating a later infection due to the continuous engagement with the chronic pathogen before encountering the secondary infection.

Finding that chronic infections increase both resistance and tolerance suggests that protection may be due to multiple mechanisms and leaves a wide range of mechanisms that could be responsible for protection against secondary infections. To gain more insight, we further investigated the impact of chronic S. marcescens infection, which had a strong protective effect against secondary *P. rettgeri* infection across a 5,000-fold range of infectious doses (Abs_600nm_ 0.001 to 5.0 or approximately 20 to 10,000 CFU/fly) (Fig. 1). Chronic infection with S. marcescens even protects against the highly virulent *Drosophila* pathogen *P. sneebia* (Fig. 4). This suggests that the mechanism of protection is likely fast acting or already expressed in chronically infected flies as high doses of *P. rettgeri* and all doses of *P. sneebia* cause mortality within 24 to 48 h in control flies (Fig. 4).

One possible protective mechanism is that chronically infected flies have increased levels of antimicrobial compounds that kill the secondary bacteria as it begins to replicate. While antimicrobial peptides (AMPs) are generally thought to be a slower acting response that takes hours to affect the outcome of infection (28), chronic infection causes higher basal expression of AMPs (7). Supporting this hypothesis, S. marcescens has a stronger induction of AMPs compared to E. faecalis, which corresponds to the more robust protection provided by S. marcescens chronic infection against secondary infection (7, 9). In addition, changes in the sequence of *diptericin* significantly impact the ability of flies to survive *P. rettgeri* infection (23). Our data provide further support for this hypothesis since the lowest dose of S. marcescens did not offer significant protection against *P. rettgeri* and *P. sneebia* infection, and also had significantly lower expression of *diptericin* relative to other chronically infected flies (Fig. 5 and 6). This is not surprising, as the infectious dose of S. marcescens positively correlates with the number of bacteria present one week later (5, 7), and during infection with Listeria monocytogenes, AMP expression correlates with the number of bacteria present (17). The relevance of increased basal AMPs to survival during *P. sneebia* secondary infection is less clear, as it is unknown which AMPs are effective against *P. sneebia*. *P. sneebia* does not itself induce a strong increase of expression of AMP genes (21); however, the higher basal levels of AMP gene expression in flies chronically infected with S. marcescens may confer an advantage to the fly by delaying or decreasing the chance of mortality if these AMPs are effective at killing *P. sneebia*.

Stimulation of other innate immune responses, like melanization or phagocytosis, by chronic infection could also influence protection during secondary infection. Long-term transcriptional changes during infection with S. marcescens and E. faecalis, however, are not enriched for genes implicated in both phagocytosis and melanization (9), suggesting that these mechanisms are less likely to play a role in protection. There is evidence that phagocytosis is important for *P. rettgeri* infection, as RNAi knockdown of Tep2, a thioester-containing protein that promotes phagocytosis, results in reduced resistance to *P. rettgeri* infection (29). The role of phagocytosis and melanization in protection against secondary infection could be an interesting topic of future studies.

It is also possible that protection is due to direct bacterial competition as a result of occupying the same niche or consuming similar resources, and that having fewer bacteria during chronic infection minimizes the competition. E. faecalis, however, does not offer as robust protection as S. marcescens, even though the number of bacteria present during chronic infection is similar with flies supporting 10^4^ to 10^5^ CFU/fly seven days postinfection (7). This suggests that resource competition is less likely to be the cause of increased resistance unless there are critical metabolites consumed or locations occupied by *Serratia* and *Providencia* species but not by E. faecalis. The primary niche occupied by all three pathogens is the hemolymph and the bacteria occupy all body segments during early infection, although whether they selectively adhere to different organs and tissues through the fly is unknown (4, 21, 30). The hemolymph contains a wide range of metabolites, including the sugars glucose, maltose, and trehalose (31). In liquid culture, both S. marcescens and E. faecalis strains are typically able to ferment all three of these sugars, potentially reducing the sugars available for *P. rettgeri*, which ferments glucose, and *P. sneebia*, which ferments both glucose and trehalose (32–34). As both species used to induce chronic infection could consume the three sugars present in the hemolymph, the competition over sugar resources does not explain the stronger protective effect of S. marcescens.

Iron is another key resource that could influence the ability of bacteria to replicate, and increased competition for this key resource could influence the ability of the secondary infection to replicate. Not only could the chronically infecting bacteria be using the available iron, but the host may also be further restricting access to iron. Infection with other bacterial pathogens (e.g., Pseudomonas entomophila and Micrococcus luteus) lowers the iron concentration in the hemolymph and increases sequestration of iron in the fat body (35). While reduction of iron in the hemolymph has not specifically been demonstrated for the pathogens used to establish chronic infections in our study, there is evidence of altered iron metabolism during these infections. A heme protein (CG1358) is upregulated during chronic infection with S. marcescens and a regulator of iron homeostasis (*Tsf1*) is also upregulated during chronic infection with both S. marcescens and E. faecalis, potentially changing the availability of iron for the secondary infection (9).

Regardless of whether increased resistance is due to increased AMP expression, competition for resources, or direct pathogen interaction, there is potentially a limit to this protection, as flies receiving the highest infectious dose of S. marcescens to induce chronic infection had variable protection and in one instance more mortality than control flies (Fig. 5A, Table S4). This suggests that there may be a concrete cost to chronically carrying such a high bacterial load from the first infection, which could be directly tested in future studies by initiating chronic infection with even higher doses. However, further exploration with higher doses must proceed with caution as these doses can cause high mortality, potentially selecting for subsets of flies with more robust immune systems (7). Selection due to primary infection likely had a minimal impact on the majority of experiments in this study in which chronic infection was induced with a moderate dose (approximately 3,000 CFU/fly). This dose caused minimal mortality from the primary infection (<25%) while delivering robust protection during secondary infection with 75% of flies surviving doses that killed all control flies (Fig. 1C–F). However, the highest doses of S. marcescens used to initiate chronic infection (approximately 6× 10^4^ CFU/fly) sometimes caused as much as 70% mortality prior to secondary infection, and this selection may have contributed to the inconsistent protection against secondary infection at this dose (Fig. 5A). Future studies that initiate chronic infection with high doses will need to account for this confounding factor.

Neither increased basal levels of AMPs or direct bacterial competition can explain the increase in tolerance that is seen in the chronically infected flies. This increase in tolerance suggests that additional mechanisms may be involved in producing the protective effect of chronic infections. One possibility is that chronically infected flies have increased negative regulation of immune responses that are either less helpful or potentially damaging to the host. Infection with S. marcescens and E. faecalis both cause sustained upregulation of a key negative regulator, Peptidoglycan recognition protein (PGRP)-LB, an amidase that degrades bacterial Dap-type peptidoglycan, which is produced by both S. marcescens and *P. rettgeri* (9, 36). Degradation of bacterial peptidoglycan helps control the level of immune stimulation through the *imd* pathway (36–38). PGRP-LB is necessary to prevent immunopathology due to innocuous gut bacteria (37). Chronic infection with S. marcescens also has continued upregulation of another catalytic PGRP protein that degrades Dap-type peptidoglycan, PGRP-SB1, which could potentially explain the larger effect of S. marcescens chronic infection on tolerance (Fig. 3) (9, 38). If chronically infected flies have a greater capability to degrade peptidoglycan, they may avoid unnecessary overstimulation of the immune response, which may prevent additional stress on the fat body and unnecessary consumption of resources.

Another possibility is that chronically infected flies have specifically shifted their cellular processes to become more tolerant of the fat body ER stress elicited in response to infection. The fat body is responsible for producing many immune effectors in response to infection, putting a heavy translational burden on the tissue. CrebA is a key regulator of ER stress and infection with both S. marcescens and E. faecalis elicit long term upregulation of this gene (9). In addition, loss of CrebA results in reduced tolerance of *P. rettgeri* infection (9). This suggests that upregulation of CrebA during chronic infection could be providing increased tolerance during secondary infection.

Improved tolerance could also be due to differences in other processes as there are many other genes that are altered during chronic infection, especially in the case of S. marcescens (8). Both *Drosophila* and mouse studies indicate that regulation of metabolism could potentially be responsible for improved tolerance to secondary infection (39–43). The protective effect observed here in D. melanogaster is reminiscent of the protection offered by gut colonization by Escherichia coli 21:H in mice, which improves tolerance to later infection with Salmonella Typhimurium or Burkholderia thailandensis by inflammasome-dependent reduction of wasting (40). There is some direct evidence that S. marcescens chronic infection could modulate metabolism. CrebA, which remains significantly upregulated during S. marcescens infection, is not only responsible for modulating fat body stress, but also modulates metabolism (9). In addition, the upregulation of genes important in iron homeostasis during chronic infection could not only improve resistance but also tolerance, as increasing the amount of iron sequestering proteins improved tolerance during sepsis by modulated gluconeogenesis in mice (44). Infection with S. marcescens also directly causes downregulation of genes involved in carbohydrate metabolism, however, many of these changes in expression rebound by 5 days postinfection (9). Altogether this suggests that altering host metabolism either through genetics or environment may alter the protective effect offered by chronic infection.

One limitation of this work is that all experiments used laboratory-housed male fruit flies. In future studies, it will be important to test the impact of chronic infection in females and flies in more natural settings. The immune response to these bacterial pathogens is sexually dimorphic in D. melanogaster with females being more susceptible to both *P. rettgeri* and E. faecalis, two of the pathogens in our study (45). Not only could this impact the ability of female flies to survive and sustain chronic infection, but differences in resource allocation due to reproduction could affect investment in resistance and tolerance during a secondary infection. Similarly, the energetic demands of fruit flies in their natural setting could be more intense, and whether chronic infection influences fitness in the wild is unknown.

This work establishes a coinfection system with a strong protective phenotype, especially in the case of S. marcescens, that will enable future testing of the role of specific genes in both tolerance and resistance during secondary infection. In addition to improving our basic understanding of how two infections interact in a host, learning the mechanisms responsible for improved tolerance in chronically infected flies could suggest new therapeutic strategies for improving tolerance.

## MATERIALS AND METHODS

### *Drosophila* husbandry.

For all experiments, wild-type Canton S Drosophila melanogaster (BDSC stock no. 1, obtained from the Lazzaro lab at Cornell University) were used. To rear age-matched adults, 10 males and 10 females were placed into plastic vials with approximately 5 mL of Nutri-fly Bloomington formulation fly food (Genesee Scientific, San Diego, CA). To prevent fungal and microbial growth, 4.8 mL of 1 M propionic acid and 1 g Tegosept (Genesee Scientific, San Diego CA) suspended in 10 mL of ethanol were added to 1 L of prepared food after the food cooled to approximately 40°C. Flies laid eggs for two days before adult flies were emptied from the vials. Ten days after adult flies were removed, newly eclosed adult flies (0 to 2 days old) were sorted in vials of 10 males. These flies were housed for an additional 5 days before infection procedures to allow their immune systems to fully develop so that experiments were conducted with 5 to 7-day-old adult flies. Throughout rearing and experiments, all flies were kept in a 25°C incubator with a 12 h light-dark cycle.

### Bacterial strains and preparation.

Four bacterial species were used, three of which were isolated from hemolymph extracted from D. melanogaster caught in State College, PA, Providencia rettgeri (strain Dmel), Providencia sneebia, and Enterococcus faecalis (32), and Serratia marcescens (strain BPL), which is an attenuated strain derived from the type strain ATCC 13880 (46).

Long term bacterial stocks were stored in 15% glycerol at −80°C and were initially grown by quadrant streaking on Luria-Bertani (LB) agar plates. These plates were incubated overnight at 37°C and then were stored at 4°C for up to 1 month. The evening before an infection experiment, an isolated colony from a streak plate was used to make an overnight culture. *P. rettgeri*, S. marcescens, and *P. sneebia* cultures were prepared in 4 mL of LB broth and incubated at 37°C with shaking at 270 rpm in a VWR incubating mini shaker. The shaking allows for optimal growth as it introduces more oxygen to the bacteria. E. faecalis cultures were prepared with 4 mL of brain and heart infusion broth (BHI) and incubated at 37°C without shaking, as they grow optimally in low oxygen environments. The following day, the bacteria were centrifuged for 10 min at 10,000 × *g* and excess media removed. The bacteria were then resuspended and diluted using Phosphate Buffer Solution (PBS) to a desired optical density at 600 nm (Abs_600nm_).

### *Drosophila* injection.

Injections were performed using a Nanoject III (Drummond Scientific, Broomall, PA) as described previously by Khalil et al. (47). On the day of injection, a glass capillary tube was pulled using a vertical micropipette puller (Sutter Instruments P-30) to create a glass needle and then back filled with mineral oil and loaded onto the Nanoject III. The needle was then partially emptied and refilled with the desired injection solution. Flies were anesthetized with CO_2_ and were then injected with 20 nL at a rate of 100 nL per second. Flies were anesthetized for less than 5 min and were returned to the 25°C incubator immediately after injection. Chronic infections were established in 5 to 7-day-old males via abdomen injection, as these have low mortality and allow large numbers of flies to survive to one week postinfection (30). Secondary infections were delivered through thorax injection to generate infections with higher mortality rates (30).

Infectious dose was confirmed by determining the CFU injected into the fly. For each dose, either two flies were individually homogenized in 100 μL of PBS immediately after injection or 20 nL of the bacterial solution was injected into 100 μL of PBS. These solutions were then spot-plated or spiral-plated using a Don Whitley automated spiral plater as described in Khalil et al. (47). After one night in an incubator, the plates were either manually counted or read using a PROtocol colony counter to determine the number of CFU injected for each dose.

### Experimental conditions.

In order to determine whether chronic infection with S. marcescens and E. faecalis protected against secondary infection with *P. rettgeri* at a range of doses, 5 to 7-day-old males were first injected in the abdomen with PBS or a bacteria diluted to 0.1 Abs_600nm_, which resulted in about 3,000 CFU per fly for S. marcescens and 2,000 CFU per fly for E. faecalis to initiate chronic infection. These doses resulted in less than 25% mortality in the first week after infection for any given independent injection, consistent with previous abdomen injections of these pathogens (7). After one week, surviving flies were given a secondary infection with different doses of *P. rettgeri*, ranging from 0.001 to 5.0 A_600nm_, which resulted in approximately 20 to 10^5^ CFU per fly. Survival was recorded daily for at least three days post-secondary infection as most deaths due to *P. rettgeri* infection occur in the first three days (5, 21, 22, 30). These experiments were divided into blocks where 12 to 28 vials of 10 male flies were injected on any given date, with 20 to 30 males per condition within a given date. To ensure that fly mortality plateaued as expected, a subset of blocks were monitored for a full week post-secondary infection. As only flies that survived the first infection were given a secondary injection, the final number of flies in our analysis is sometimes lower due to mortality from the primary infection. To investigate how chronic infection with S. marcescens and E. faecalis impacted resistance and tolerance, bacterial load of *P. rettgeri* was assessed 10 h post-secondary infection for a subset of experiments described above.

In order to determine whether chronic infection with S. marcescens protected against *P. sneebia* secondary infection, 5 to 7-day-old males were first injected in the abdomen with PBS or S. marcescens diluted to 0.1 Abs_600nm_, which results in approximately 3× 10^3^ CFU per fly, to initiate chronic infection. After 1 week, surviving flies were given a secondary infection with different doses of *P. sneebia*, ranging from 0.001 to 1.0 Abs_600nm_, which resulted in approximately 10 to 10^4^ CFU per fly. Due to the high lethality of *P. sneebia* survival was recorded every 2 h from 18 to 48 h post-secondary infection. The experiment was divided into blocks where 24 to 28 vials of 10 male flies were injected on any given date, with approximately 20 to 30 males per condition.

In order to assess the impact of chronic infection dose on protection granted by chronic infection, 5 to 7-day-old males were first injected in the abdomen with PBS or one of four doses of S. marcescens ranging from 0.001 to 2.0 Abs_600nm_ to initiate chronic infection which resulted in approximately 30 to 6× 10^4^ CFU per fly. While there was minimal mortality for the lowest doses, the highest doses caused mortality consistent with previous research with the highest dose sometimes killing up to 70% of infected flies (7). After 1 week, surviving flies were given a secondary infection with either *P. rettgeri* diluted to 2.0 Abs_600nm_ or *P. sneebia* diluted to 0.01 Abs_600nm_, which resulted in approximately 4× 10^4^ CFU/fly and 100 CFU/fly, respectively. For *P. rettgeri* secondary infection, survival was recorded daily for one week post-secondary infection. Due to the high lethality of *P. sneebia*, survival was recorded every 2 h from 18 to 48 h post-secondary infection. These experiments were divided into blocks where 20 to 28 vials of 10 male flies were injected on any given date, with approximately 40 to 50 males per condition.

### Determining bacterial load post-secondary infection.

Since two bacterial species were present, a straight CFU (CFU assay) was not possible. For flies chronically infected with E. faecalis, bacterial load was determined using a CFU assay with antibiotic selection to selectively kill E. faecalis and quantify *P. rettgeri* loads. In short, a fly was individually homogenized in 100 μL of PBS and plated using a Don Whitley automated spiral plater and LB agar plates supplemented with the antibiotic vancomycin (5 g/mL) to selectively kill E. faecalis but not *P. rettgeri*. For infectious doses above 1.0 Abs_600nm_ 1:100 dilutions were performed in PBS to prevent overgrowth on the plates. After 16 to 24 h incubating at 37°C, the plates were read using a PROtocol colony counter to determine the number of CFU per fly.

As we could not identify an antibiotic that would inhibit all S. marcescens colonies but not influence the number of *P. rettgeri* colonies, we used qPCR in order to quantify the amount of *P. rettgeri* for these dual infections. First, five flies were homogenized in 180 μL of PBS and then DNA was extracted using a Qiagen blood and tissue DNEasy kit, following the protocol for insect tissue and bacterial extraction with special attention paid to thorough homogenization and vortexing between steps. Sufficient DNA was recovered with a single elution. The quality and quantity of the DNA was tested with a Nanodrop One machine.

Using a Roche LightCycler and FastStart Essential DNA green master mix, qPCR was used to quantify the amount of a *P. rettgeri*-specific gene in comparison with the *Drosophila* gene Rp49. DNA samples were diluted 1:10 in RNase free water and each sample was run in triplicate for each primer. A standard curve was prepared with a pooled sample of all of the highest concentrations of *P. rettgeri* (conditions with the highest bacterial doses) in order to assess the efficiency of the primers under our conditions. The *P. rettgeri* primers were created using NCBI Primer BLAST for pPRET-1 and tested on DNA isolated from flies injected with a range of *P. rettgeri* suspensions to ensure that there was a linear relationship between the amplified product and the amount of *P. rettgeri* in the flies as experimentally determined by CFU assays. Rp49 primers were based on previously published primers in D. melanogaster (7). Primer sequences are reported in Table 2.

**TABLE 2:** Primer sequences used for qPCR

Gene	Forward	Reverse
*Rp49*	5′-AGGCCCAAGATCGTGAAGAA-3′	5′-GACGCACTCTGTTGTCGATACC-3′
*pPRET*	5′-AGCCGATCAGTTTGAGCGAA-3′	5′-TGTTCAATTCTCGGGGGAGC-3′
*diptericin A*	5′-GCGGCGATGGTTTTGG-3′	5′-CGCTGGTCCACACCTTCTG-3′

To convert qPCR *C_T_* values to bacterial load equivalents, the *C_T_* values for both from three technical replicates were averaged for each sample. The relative amount of *P. rettgeri* in each sample was calculated using the Pfaffl method using flies given only a secondary infection (Abs_600nm_ 0.1) at 0 h post-secondary infection as the baseline condition and Rp49 as the control gene. We experimentally determined that on average, flies injected with 20 nL of *P.rettgeri* (Abs_600nm_ 0.1), which was our baseline condition for analysis, have approximately 2,000 CFU/fly. We therefore multiplied all values by 2,000 to obtain approximate CFU equivalents for all of our samples.

### qRT-PCR to determine *diptericin* expression.

One week after initiating the chronic infection, eight flies from each condition were homogenized in TRIzol and stored at −80°C. Four experimental blocks were completed, each with a single homogenate for each condition. RNA was isolated using a standard TRIzol extraction and then treated with DNase I (Promega) to remove any contaminating DNA. One-step qRT-PCR was performed using the Luna Universal One Step RT-qPCR kit on the Roche Lightcycler, following manufacturer instructions scaled down to 15 μL reactions. Primer sequences are reported in Table 2. Relative expression was determined using the ΔΔCt method using Rp49 as a reference gene and sterile saline injected flies as the baseline condition (48), as experimentally determined primer efficiencies using pooled RNA were close to 100% (*Rp49*: 105%, *diptericin A*: 97%), which makes the assumption of doubling every cycle reasonable.

### Statistical analysis.

**(i) Survival analysis.** Survival curves were graphed in R as Kaplan-Meyer plots. While experiments were initially analyzed in R using Cox Proportional Hazards mixed effects model (*coxme*) with date of injection and primary injection dose as fixed effects and random effect terms accounting for experimental structure (e.g., date of injection, housing of groups of flies in vials), these models all violated the assumptions of Cox Proportional Hazards. Therefore, we switched to using log-rank pairwise comparisons to determine whether a given condition was significantly different compared to the control flies given only a secondary infection. Log-rank tests were performed on data from each individual date, and if experiments on all dates showed higher survival in conditions with chronic infection, *P* values were combined using Fisher’s method and only the final *P* value is reported in the text. If there was a variable effect of chronic infection, the number of individual experiments with statistically significant effects is reported.

**(ii) Resistance.** Bacterial loads were graphed in R using the package *ggplot2*. To determine whether chronic infection status significantly altered resistance to infection, we built a linear model using the lm() command in R with the log_10_(CFU/fly) or log_10_(bacterial load equivalents) at 10 h postinfection as the response variable. Factors included were block (B), dose of secondary infection (DS), and chronic infection status (C) and the interaction between dose of secondary infection and chronic infection (DS × C). Infectious dose was treated as categorical in the model as the relationship between dose and log_10_ (bacterial load) at 10 h postinfection was not linear.

Model A: log_10_(CFU/fly) or log_10_(bacterial load equivalent) = B + DS + C + (DS × C).

**(iii) Tolerance.** To determine whether chronic infection status significantly altered tolerance to infection, a multiple logistic regression model was constructed using survival at 3 days postinfection as the response variable. Explanatory variables included log_10_ (CFU/fly) or log_10_(bacterial load equivalents) (BL), chronic infection status (C), and the interaction between the two (BL × C).

Model B: Survival 3 days postinfection = BL + C + (BL × C).

The main effect of chronic infection status (C) determined whether the logistic curve significantly shifts to the left or right relative to control flies, while the interaction term (BL x C) determined whether the slope of the logistic regression differed in chronically infected flies. If the full model revealed that the interaction term was not significant, then a reduced model was fitted in order to better detect shifts in the main effect. Final models were graphed using R (*ggplot2*). The point of inflection (the log_10_ [bacterial load] where the probability of survival is 50% at 3 days post-secondary infection) and the Hill slope (the slope at the point of inflection) were calculated based on these models.

**(iv) *Diptericin* expression.** Expression data were graphed in R using the package *ggplot2*. To determine whether infectious doses of S. marcescens statistically significantly impacted diptericin expression, we conducted an ANOVA using the aov() command in R with the -ΔΔCt as the response variable. Factors included were block (B) and dose of primary infection (DP). All factors were treated as categorical in the model as the relationship between dose and *diptericin* expression in chronically infected flies was not linear. Statistically significant differences between individual doses were determined using the Tukey Test.

### Data availability.

All data and R scripts used to analyze the data are available on Github (https://github.com/moriacc/DrosophilaChronic_2022).
