# Supplementary material for: Impact of Chronic Infection on Resistance and Tolerance to Secondary Infection in Drosophila melanogaster
Source: Infect Immun. 2023 Feb 16;91(3):e00360-22. doi: 10.1128/iai.00360-22 (PMC10016074; doi:10.1128/iai.00360-22)
Supplement: Supplemental file 1 — Tables S1 to S6 and Fig. S1. Download iai.00360-22-s0001.pdf, PDF file, 0.2 MB [file iai.00360-22-s0001.pdf]

Table S1. P-values for log rank tests on individual doses within each experiment - Impact of *S. marcescens* chronic infection on varying doses of secondary *P. rettgeri* infection

	Infectious Dose (Abs600nm)					
	0.01	0.1	1	2	3	5
**Individual Blocks**						
Block 1 - August 2, 2018	0.074	0.127	0.021	–	–	–
Block 2 - August 6, 2018	0.018	0.001	0.027	–	–	–
Block 3 - June 14, 2019	0.065	<0.001	<0.001	<0.001	–	–
Block 4 - June 17, 2019	0.185	0.508	<0.001	<0.001	–	–
Block 5 - June 19, 2019	0.205	<0.001	0.003	<0.001	–	–
Block 6 - June 25, 2019	0.006	<0.001	<0.001	<0.001	–	–
Block 7 - June 27, 2019	0.650	0.008	<0.001	<0.001	–	–
Block 8 - January 30, 2020	–	<0.001	<0.001	<0.001	–	<0.001
Block 9 - February 13, 2020	–	<0.001	<0.001	<0.001	–	<0.001
Block 10 - September 27, 2020	–	–	<0.001	<0.001	<0.001	<0.001
Block 11 - October 6, 2020	–	–	<0.001	<0.001	<0.001	<0.001
Block 12 - November 1, 2020	–	–	<0.001	<0.001	<0.001	<0.001
Block 13 - June 16, 2021	–	<0.001	<0.001	<0.001	–	<0.001
**Fisher's Combined**						
Final P-value	<0.001	<0.001	<0.001	<0.001	<0.001	<0.001

Table S2. P-values for log-rank tests on individual doses within each experiment - Impact of *E. faecalis* chronic infection on varying doses of secondary *P. rettgeri* infection

	Infectious Dose (Abs600nm)			
	0.001	0.01	0.1	1
**Individual Blocks**				
Block 1 - June 11, 2018	–	0.004	0.015	0.023
Block 2 - June 22, 2018	0.315	0.014	0.005	0.018
Block 3 - June 25, 2018	0.077	0.007	0.845	0.628
Block 4 - July 19, 2018	0.725	0.987	0.702	0.864
Block 5 - June 3, 2019	0.239	0.129	0.479	0.012
Block 6 - June 5, 2019	0.010	0.184	0.620	0.872
Block 7 - July 1, 2019	–	0.007	0.845	0.628
**Fisher's Combined**				
Final P-value	0.028	<0.001	0.064	0.020

Table S3. Output of full logistic regression model for impact of bacterial load and chronic infection with *E. faecalis* on survival three days post-secondary infection

**Factor**[a]	**estimate**	**z-value**	***P***
(intercept)	2.28	4.42	<**0.001**
$\log_{10}$(bacterial load)	-0.62	-6.45	<**0.001**
chronic infection	0.17	0.24	0.81
$\log_{10}$(bacterial load):chronic infection	0.08	0.64	0.52

[a] interactions are indicated by a colon between

Table S4. P-values for log-rank tests on individual doses within each experiment - Impact of *S. marcescens* chronic infection on varying doses of secondary *P. sneebia* infection

	Infectious Dose (Abs600nm)			
	0.001	0.01	0.1	1
**Individual Blocks**				
Block 1 - October 1, 2021	0.287	<0.001	<0.001	<0.001
Block 2 - October 10, 2021	0.001	<0.001	<0.001	<0.001
Block 3 - October 24, 2021	0.002	<0.001	<0.001	<0.001
**Fisher's Combined**				
Final P-value	<0.001	<0.001	<0.001	<0.001

Table S5. P-values for log-rank tests on individual doses within each experiment - Impact of *S. marcescens* infectious dose on secondary *P. rettgeri* infection

	Primary Infectious Dose (Abs600nm)				
	0.001	0.01	0.1	1	2
**Individual Blocks**					
Block 1 - October 18, 2020	0.072	0.293	<0.001	0.070	0.041
Block 2 - October 20, 2020	0.526	0.211	<0.001	0.002	0.072
Block 3 - October 29, 2020	0.628	0.018	<0.001	<0.001	0.012
Block 4 - November 2, 2020	0.942	0.009	<0.001	<0.001	0.407
Block 5 - November 7, 2020	0.693	0.332	<0.001	<0.001	0.010
Block 6 - November 28, 2020	0.064	0.913	<0.001	<0.001	0.033
Block 7 - December 4, 2020	0.968	0.007	<0.001	<0.001	–
Block 8 - June 17, 2021	0.726	0.063	<0.001	<0.001	<0.001
Block 9 - June 18, 2021	0.238	0.001	<0.001	<0.001	<0.001
**Fisher's Combined**					
Final P-value	0.733	<0.001	<0.001	<0.001	N.C.

Table S6. P-values for log-rank tests on individual doses within each experiment - Impact of *S. marcescens* infectious dose on secondary *P. sneebia* infection

	Primary Infectious Dose (Abs600nm)				
	0.001	0.01	0.1	1	2
**Individual Blocks**					
Block 1 - March 10, 2021	0.029	0.076	<0.001	0.700	0.628
Block 2 - March 17, 2021	0.689	0.048	<0.001	0.002	–
Block 3 - March 31, 2021	0.536	0.842	0.478	<0.001	<0.001
Block 4 - April 21, 2021	0.011	<0.001	<0.001	<0.001	<0.001
Block 5 - April 28, 2021	0.958	0.868	0.009	<0.001	<0.001
Block 6 - June 20, 2021	0.509	0.032	<0.001	<0.001	<0.001
**Fisher's Combined**					
Final P-value	0.261	<0.001	<0.001	<0.001	<0.001

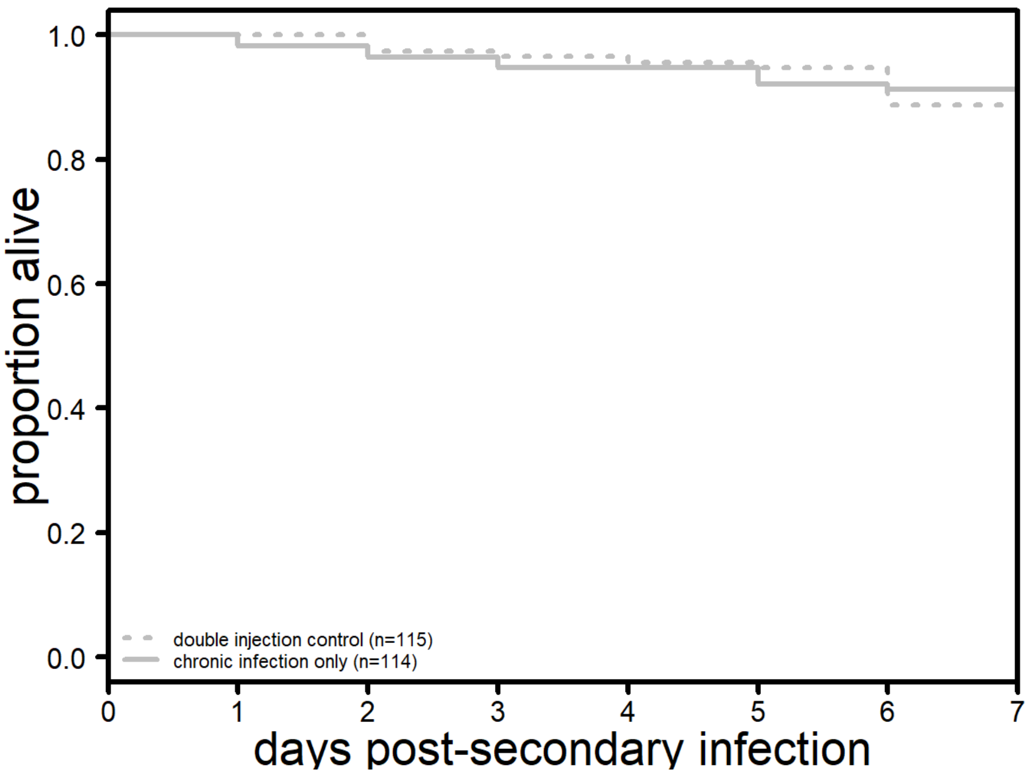

Figure S1. Survival of control conditions. Five to seven day old flies were injected with sterile PBS or a bacterial suspension of *S. marcescens* (Abs_{600nm} 0.1) in the abdomen and then one week later injected with sterile PBS in the thorax. Graphs show combined mortality data for all experimental dates and total number of flies per condition across all dates is indicated in the legends.
